# Pre-stimulus bioelectrical activity in light-adapted ERG under blue versus white background

**DOI:** 10.1017/S0952523823000032

**Published:** 2023-12-13

**Authors:** Katherine Tsay, Sara Safari, Abdullah Abou-Samra, Jan Kremers, Radouil Tzekov

**Affiliations:** 1 Morsani College of Medicine, University of South Florida, Tampa, FL, USA; 2 University Hospital Erlangen, Erlangen, Germany; 3 Department of Ophthalmology, University of South Florida, Tampa, FL, USA; 4 Department of Medical Engineering, University of South Florida, Tampa, FL, USA

**Keywords:** eye, retina, electroretinography, photopic negative response, ERG, PhNR

## Abstract

To compare the baseline signal between two conditions used to generate the photopic negative response (PhNR) of the full-field electroretinogram (ERG): red flash on a blue background (RoB) and white flash on a white background (LA3). The secondary purpose is to identify how the level of pre-stimulus signal affects obtaining an unambiguous PhNR component. A retrospective chart review was conducted on four cohorts of patients undergoing routine ERG testing. In each group, LA3 was recorded the same way while RoB was generated differently using various luminances of red and blue light. The background bioelectrical activity 30 ms before the flash was extracted, and the root mean square (RMS) of the signal was calculated and compared between RoB and LA3 using Wilcoxon test. Pre-stimulus noise was significantly higher under RoB stimulation versus LA3 in all four conditions for both right and left eyes (ratio RoB/LA3 RMS 1.70 and 1.57 respectively, *p* < 0.033). There was also no significant difference between the RMS of either LA3 or RoB across protocols, indicating that the baseline noise across cohorts were comparable. Additionally, pre-stimulus noise was higher in signals where PhNR was not clearly identifiable as an ERG component versus signals with the presence of unambiguous PhNR component under RoB in all four groups for both eyes (*p* < 0.05), whereas the difference under LA3 was less pronounced. Our study suggests that LA3 produces less background bioelectrical activity, likely due to decreased facial muscle activity. As it seems that the pre-stimulus signal level affects PhNR recordability, LA3 may also produce a better-quality signal compared to RoB. Therefore, until conditions for a comparable bioelectrical activity under RoB are established, we believe that LA3 should be considered at least as a supplementary method to evaluate retinal ganglion cell function by ERG.

## Introduction

The photopic negative response (PhNR) is a slow negative component of the photopic electroretinogram (ERG) that is specific in evaluating the activity of retinal ganglion cells (Viswanathan et al., 1999). According to the extended protocol for the PhNR of the full-field electroretinogram from the International Society for Clinical Electrophysiology of Vision (ISCEV) (Frishman et al., 2018), the preferred method of eliciting a PhNR is a red flash on a rod-saturating blue background (RoB). However, a PhNR can also be elicited by a white flash on a white background, under stimulus conditions like the one used to elicit an ISCEV standard Light-adapted 3 ERG (LA3) response (McCulloch et al., 2015; Robson et al., 2022).

Although many studies have recorded the PhNR under RoB and white flash on a white background separately, very few studies have used both conditions on the same subjects or the same clinical population. Even fewer studies have provided a quantitative analysis of the PhNR amplitude recorded under both conditions and the results were contradictory. Sustar et al. (2009) reported 25–29% higher PhNR amplitude recorded under RoB in their two control groups (n = 20; n = 21), while Banerjee et al. (2019) reported 15–20% lower PhNR amplitude under RoB in two control groups (n = 20; n = 50) compared to white on white. In both cases, the responses were recorded using 10 cd/m^2^ blue or white background; Sustar et al. used flash stimuli ranging from 0.08 to 7.5 cd**⋅**s/m^2^, whereas Banerjee et al. used a 3.5 cd**⋅**s/m^2^ stimulus strength for red and white flash. A third report, by Shen et al. (2013) using a larger control group (n = 36), showed only a 5% difference in amplitude in favor of the RoB condition using white and red stimuli of 2 cd**⋅**s/m^2^ on white and blue background of 25 cd/m^2^. Kremers et al. (2012) showed no difference in amplitude between PhNR recorded under RoB or white flash on a white background in healthy volunteers (n = 14) when flash and background are matched according to the photopic luminous efficiency function (V_λ_). Finally, Rangaswamy et al. (2007) reported larger PhNR amplitudes under RoB conditions (0.04 to 2.84 cd**⋅**s/m^2^ red flash on 10 cd/m^2^ blue background) compared to white on white conditions (0.04 to 2.84 cd**⋅**s/m^2^ white flash on 40 cd/m^2^ white background) in anesthetized monkeys. In glaucoma patient populations, the differences were more pronounced, but also varied: −33% (Robson et al., 2022), −47% (Banerjee et al., 2019), and + 3% (Shen et al., 2013), suggestive of dependence on various factors like recording instrumentation, patient population demographics, and selection criteria, etc.

As amplitude and timing of PhNR determination depends heavily on the identification of the PhNR trough after the b-wave, clear and unambiguous detection of this feature of the ERG waveform is needed. In turn, when comparing the results between the two conditions (e.g. RoB versus LA3), both the amplitude and the signal-to-noise ratio (SNR) are of importance. Therefore, an estimate of noise would be helpful for results obtained under the two conditions.

The purpose of the current study was to compare the pre-stimulus bioelectrical activity as a source of noise between the two conditions in patients with various retinal pathologies. The secondary purpose was to identify how the level of noise in the pre-stimulus signal would affect the recordability of the PhNR. Blue backgrounds are perceived to be brighter than white backgrounds of equal luminance thereby producing more visual discomfort. The present study provides insight in how visual comfort influences the quality of ERG signals in patients.

## Materials and methods

### Standard clinical ERG procedure

Four cohorts of participants (Groups 1–4) underwent a standard clinical ERG (McCulloch et al., 2015) at the University of South Florida Eye Institute in Tampa, Florida between July 2016 and October 2021. The protocol defined as “Light-adapted 3 ERG” was used to elicit one type of PhNR response in our study, referred to as LA3. In addition, after the standard ERG recordings, a series of additional responses were obtained, including the RoB response.

As per ISCEV protocol, the participants underwent 10 min of light adaptation before recording LA3 ERGs. That was followed by recording a 30 Hz flicker and other protocols using standard white background before recording RoB. For RoB recording a shorter adaptation time (30 sec–2 min) was used for the blue background illumination before proceeding with RoB stimulation. The decision to use a relatively shorter adaptation time for the blue background was determined by the understanding that the time course of chromatic adaptation in light-adapted eyes is a relatively quick process essentially completed in 2 min (Rinner & Gegenfurtner, 2000).

Groups 1–3 were recorded after June 2018 using the UTAS SunBurst system, while Group 4 was recorded before June 2018 using the UTAS E-3000 system, both from LKC Technologies (Gaithersburg, MD). In all groups, the filter bandwidth for recording both LA3 and RoB recordings was 0.3–500 Hz and a notch filter was not used. Recording was done using DTL electrodes as active and single use Ag/AgCl electrodes as reference, the latter residing on the cheekbones; ground electrodes were also single-use Ag/AgCl placed over the left mastoid. The sampling rate was either 2004 Hz, 3340 Hz, or 3757 Hz and was always taken into account when calculating the magnitude of the pre-stimulus signal and doing digital filtering. Of note, a 30 ms pre-stimulus period was used in all recordings. All data were collected by the same experienced operator (RT).

### Protocols

Throughout the four groups, LA3 was recorded in the same way (2.5 cd**⋅**s/m^2^ white flash on a 30 cd/m^2^ white background), while the RoB response was generated differently in each group:

- Group 1 (Gr1) RoB protocol used a 5 cd**⋅**s/m^2^ red LED flash (λmax = 627 nm, half-amplitude bandwidth = 20 nm) on a 30 cd/m^2^ blue LED background (λmax = 470 nm, half-amplitude bandwidth = 25 nm)
- Group 2 (Gr2) protocol used a 5 cd**⋅**s/m^2^ red LED flash on a 10 cd/m^2^ blue LED background,
- Group 3 (Gr3) protocol used a 2.5 cd**⋅**s/m^2^ red LED flash on a 10 cd/m^2^ blue LED background,
- Group 4 (Gr4) protocol used a 2.5 cd**⋅**s/m^2^ red xenon flash (Wratten #25 filter) on a 28 cd/m^2^ blue halogen illumination background (Wratten #47B filter).

### Data analysis

**Figure 1.**
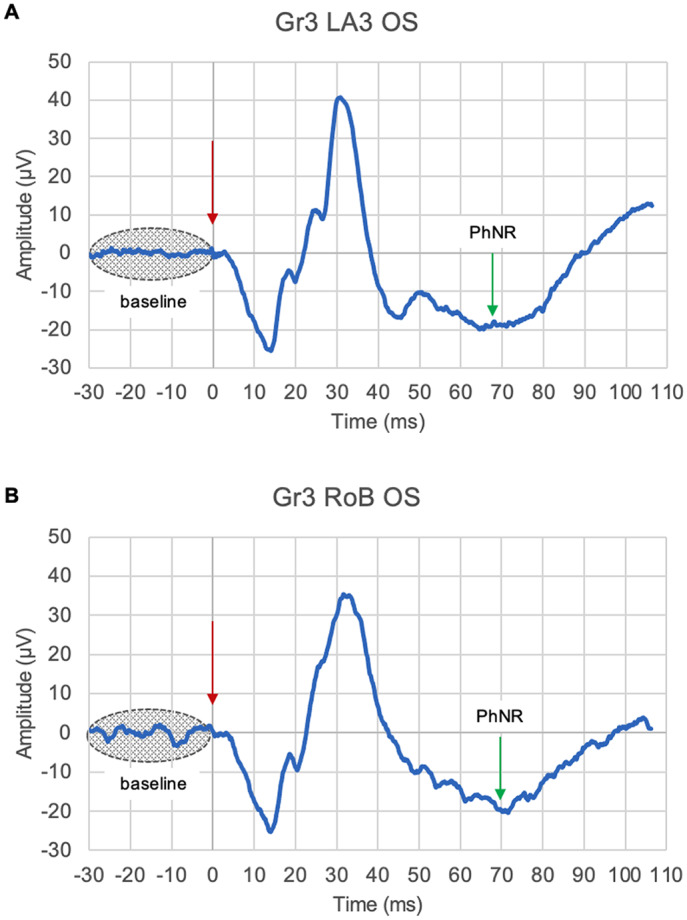
Representative traces from a patient undergoing ERG stimulation via LA3 (A) and Gr3 RoB protocol (B) of the left (OS) eye. The horizontal axis represents time in ms, where 0 ms represents the time of the flash (indicated by red arrow); Y-axis represents the amplitude of the bioelectrical signal generated by the retina in μV. Green arrow represents the PhNR.

The level of background bioelectrical activity from 30 ms before the flash to the time of the flash (Figure 1) was compared between the LA3 and RoB conditions by calculating the root mean square (RMS) of the signal under all four protocols.

A potential downside of this analysis comes from a limitation imposed by the recording equipment. The equipment used does not provide the capability of storing the number of traces used when coming up with the final averaged response for clinical reporting after removing records containing artifacts. As reconstructing the process of elimination of artifacts for all records to determine the number of signals used in the final averaged response is time consuming and resource-intensive, a different approach was used to eliminate the influence of unequal number of averaged signals. Specifically, to address this issue and also to find out where there may be a temporal variation within the course of the recording, we analyzed the pre-stimulus bioelectrical activity of individual recordings from a fixed number of recordings from each group. The number of recordings used for various protocols was either 20, 25, or, in most cases, 30. Therefore, we analyzed the first 20 individual recordings from the data sample. To minimize a potential effect of an unequal number of patients in each group, we set the sample size for each group to 13 patients, equal to the size of the smallest group (Group 1), which resulted in analyzing 52 patients’ data (104 eyes), or ~ 45% of the original sample. As some recordings showed unusually high RMS values likely due to eye movements, an outlier analysis was performed using the ROUT method in GraphPad Prism, setting the False Discovery Rate (Q) to 1%. The RMS values were averaged across all subjects and the mean values plotted against time. An ANOVA test was conducted between corresponding LA3 and RoB RMS values and additionally, a mixed-effects model (REML) analysis was performed in GraphPad Prism.

To estimate the effect of the strength of the pre-stimulus signal on the quality of the post-stimulus signal, we conducted an analysis to determine the rate of instances when the PhNR component presented itself in an unambiguous way. For this analysis, PhNR was defined as a well-formed and clearly distinguishable trough, occurring 30–40 ms after the b-wave peak, typically after an i-wave. Two independent observers evaluated the traces, and disagreements were resolved with discussion and reaching of consensus. Representative traces of signals where the PhNR trough was or was not clearly distinguishable are presented in Supplementary Figure 1.

As an additional method of analysis, a Fast Fourier Transform (FFT) was applied to the pre-stimulus part of the signal. The portion of the signal of interest (30 ms pre-flash) was extracted from the main signal, zero padded up to a length of 512 samples to allow better frequency domain resolution and windowed with a Hamming window to reduce transitioning artifacts, then subjected to FFT in Matlab (MathWorks, Natick, MA) to evaluate the frequency content. The average magnitude of the FFT was plotted against frequency. Mean values and SEM of the first 1/3 of the spectrum were plotted for right and left eyes separately. The area under the curve restricted to the average values for this part of the spectrum was calculated for the right and left eyes in GraphPad Prism.

### Statistical analysis

Normality of the data distribution was checked with D’Agostino & Pearson test. As it turned out that the data were not normally distributed, inter-group comparison was done using Wilcoxon matched-pairs singed rank test, Kruskal-Wallis test, and Brown-Forsythe ANOVA test. Comparison between LA3 and RoB signals when aggregated as raw results was done by 2-way ANOVA. Analysis to identify outliers was carried using the ROUT method in Prism with Q set to 1%. For comparison between LA3 and RoB signals after removal of outliers, a mixed-effects analysis (based on an REML model) was used, without assuming sphericity and alpha set to 0.05, followed by Šídák’s multiple comparisons test. GraphPad Prism 9.3 (GraphPad Software LLC, San Diego, CA) was used for statistical analysis and graphing.

## Results

### Participants

The medical records and ERG recordings of 117 patients/233 eyes were evaluated: Gr1 n = 13 patients/26 eyes; Gr2 n = 29/58; Gr3 n = 32/63; Gr4 n = 43/86 (Table 1). There was no statistically significant difference between the ages of the four groups (Brown-Forsythe ANOVA test, *p* = 0.8794).

**Table 1.** Summary of patient demographics

	n			Age
	Male	Female	Total	Mean ± SD
Gr1	5	8	13	51.9 ± 15.9
Gr2	7	22	29	49.4 ± 13.1
Gr3	7	25	32	50.9 ± 16.1
Gr4	17	26	43	52.4 ± 18.9
Total	**36**	**81**	**117**	**51.2 ± 16.5**

The participants had a variety of clinical preliminary diagnoses. Of the 117 patients, 52 had a diagnosis of a visual disturbance (ICD-10 codes H53 and H54), such as visual loss, visual field defects, or night blindness. 42 had a diagnosis of a retinal disorder (ICD-10 code H35), such as macular degeneration, peripheral retinal degeneration, hereditary retinal dystrophy, or pigmentary retinal dystrophy. The remaining patients had diagnoses of: disorders of the optic nerve (H46 & H47, n = 9), chorioretinal inflammation and other disorders of the choroid (H30 & H31, n = 10), of the globe (H44, n = 1), or of the vitreous body (H43, n = 1). One patient had a retinal vein occlusion (H34), and another had a benign neoplasm of the eye (H31). This work was conducted using the data collected for another study where glaucoma was an exclusion criterion, therefore it was also an exclusion criterion in our study.

As shown in Table 1, there was a sex imbalance in the study population. The male patients made up on average 31% of the whole population, and this percentage varied from 24% to 39% across the different groups.

### Baseline bioelectrical activity

**Figure 2.**
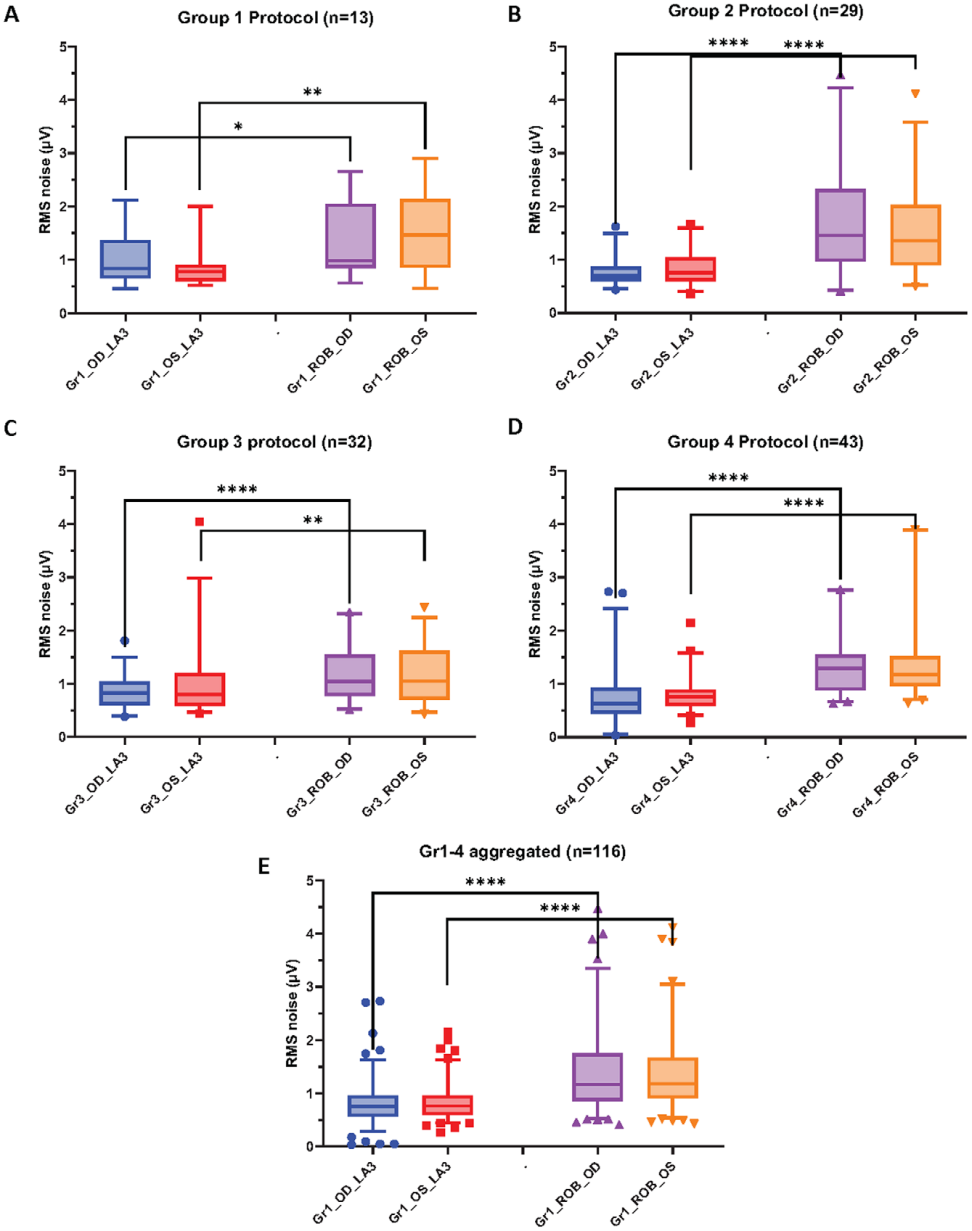
Graphical representation of mean RMS values for each group and results of statistical comparison. The first four panels show data obtained using a specific protocol (2A – Gr1; 2B – Gr2; 2C – Gr3; 2D – Gr4), whereas the last panel (2E) shows an aggregate of all participants. Boxes represent 25–75 percentile, the horizontal line within the box represents the median, the whiskers represent 5 to 95 percentiles. Statistical significance (Wilcoxon matched-pairs signed rank test): ** - *p* < 0.01; **** - *p* < 0.0001.

Overall, the paired comparison (Wilcoxon matched-pairs signed rank test) of the baseline level of bioelectrical activity showed that the RoB condition resulted in statistically significantly higher magnitude of pre-stimulus noise compared to the corresponding LA3 conditions in all four groups (Gr1: Figure 2A; Gr2: Figure 2B; Gr3: Figure 2C; Gr4: Figure 2D). This finding was true for both the right (OD) and left (OS) eyes. For Gr1 OD, the mean RMS of the baseline bioelectrical activity of the RoB condition was 1.38 while the LA3 condition was 0.99 (*p* = 0.033); for Gr1 OS, the mean RMS of RoB was 1.49 while LA3 was 0.91 (*p* = 0.001). Statistically significant differences between RoB and LA3 were found for Gr2-Gr4 as well. The comparisons of the mean RMS for each condition are summarized in Table 2. Overall, the RMS was about a factor 1.6 larger in the RoB conditions.

**Table 2.** RMS values of baseline signal comparisons between LA3 and RoB

		Gr1	Gr2	Gr3	Average Gr1–3	Gr4
LA3 OD	*RMS ± SD (μV)*	0.99 ± 0.48 (n = 13)	0.77 ± 0.26 (n = 29)	0.85 ± 0.32 (n = 32)		0.76 ± 0.31 (n = 43)
RoB OD		1.38 ± 0.70 (n = 13)	1.77 ± 1.15 (n = 29)	1.21 ± 0.58 (n = 31)		1.41 ± 0.97 (n = 43)
*p*-value^a^		0.033	<0.0001	<0.0001		<0.0001
RoB/LA3 ratio		1.39	2.30	1.42	1.70	1.86
LA3 OS	*RMS ± SD (μV)*	0.91 ± 0.47 (n = 13)	0.84 ± 0.33 (n = 29)	0.98 ± 0.67 (n = 32)		0.80 ± 0.35 (n = 43)
RoB OS		1.49 ± 0.80 (n = 13)	1.58 ± 0.87 (n = 29)	1.16 ± 0.54 (n = 31)		1.54 ± 1.19 (n = 43)
*p*-value^a^		0.001	<0.0001	0.009		<0.0001
RoB/LA3 ratio		1.64	1.88	1.18	1.57	1.93

Abbreviations: LA3, light-adapted 3 (white flash on white background); OD, right eye; OS, left eye; RMS, root mean square; RoB, red flash on blue background; SD, standard deviation.

a Wilcoxon matched-pairs signed rank test.

Of note, when comparing LA3 or ROB across protocols (e.g., Gr1 LA3 versus Gr2 LA3 versus Gr3 LA3), the baseline level of bioelectrical activity was not statistically significantly different for either the right eyes or the left eyes (Kruskal-Wallis test; multiple comparisons; *p* > 0.05); Gr4 was not included in the comparisons as the signal was recorded with different equipment. These results suggest that the baseline bioelectrical activity was comparable across all conditions.

### Equal size group analysis

**Figure 3.**
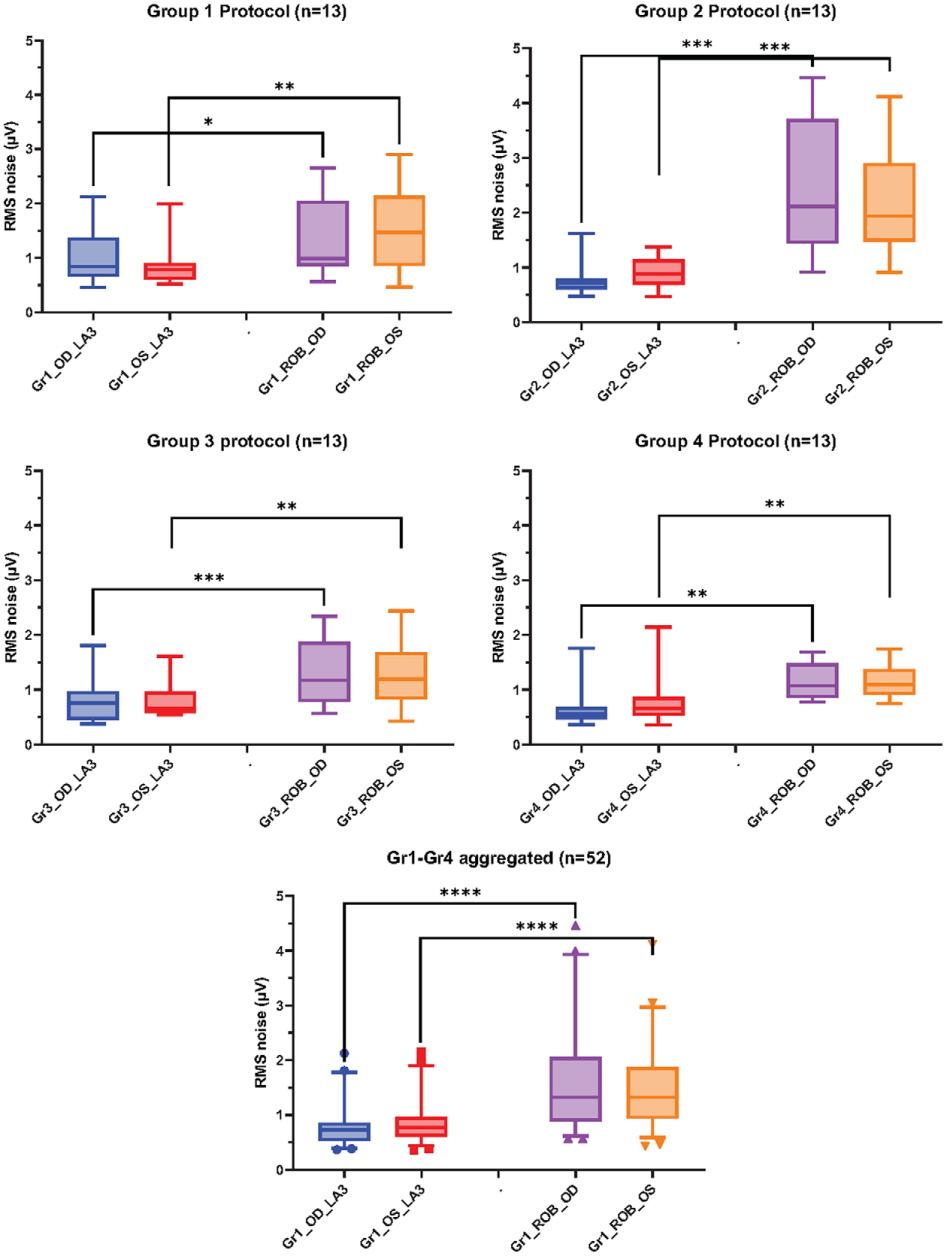
Graphical representation of mean RMS values for each group for equal size group analysis. Other designations same as in Figure 2.

To compare how the pre-stimulus noise for LA3 and RoB compared between the two recording conditions across groups with balanced number of participants, an equal sample size (n = 13 patients/group) analysis was conducted. The average pre-stimulus RMS noise for RoB was consistently and significantly higher than the one for LA3 for both right and left eyes for all four groups (*p* < 0.05), as shown in Figure 3A–D. This was also true for the aggregated data from all four groups (*p* < 0.0001), shown in Figure 3E. In the latter, the ratio of the median RMS values for RoB/LA3 of the pre-stimulus noise was 1.83 for right eyes and 1.72 for left eyes.

**Figure 4.**
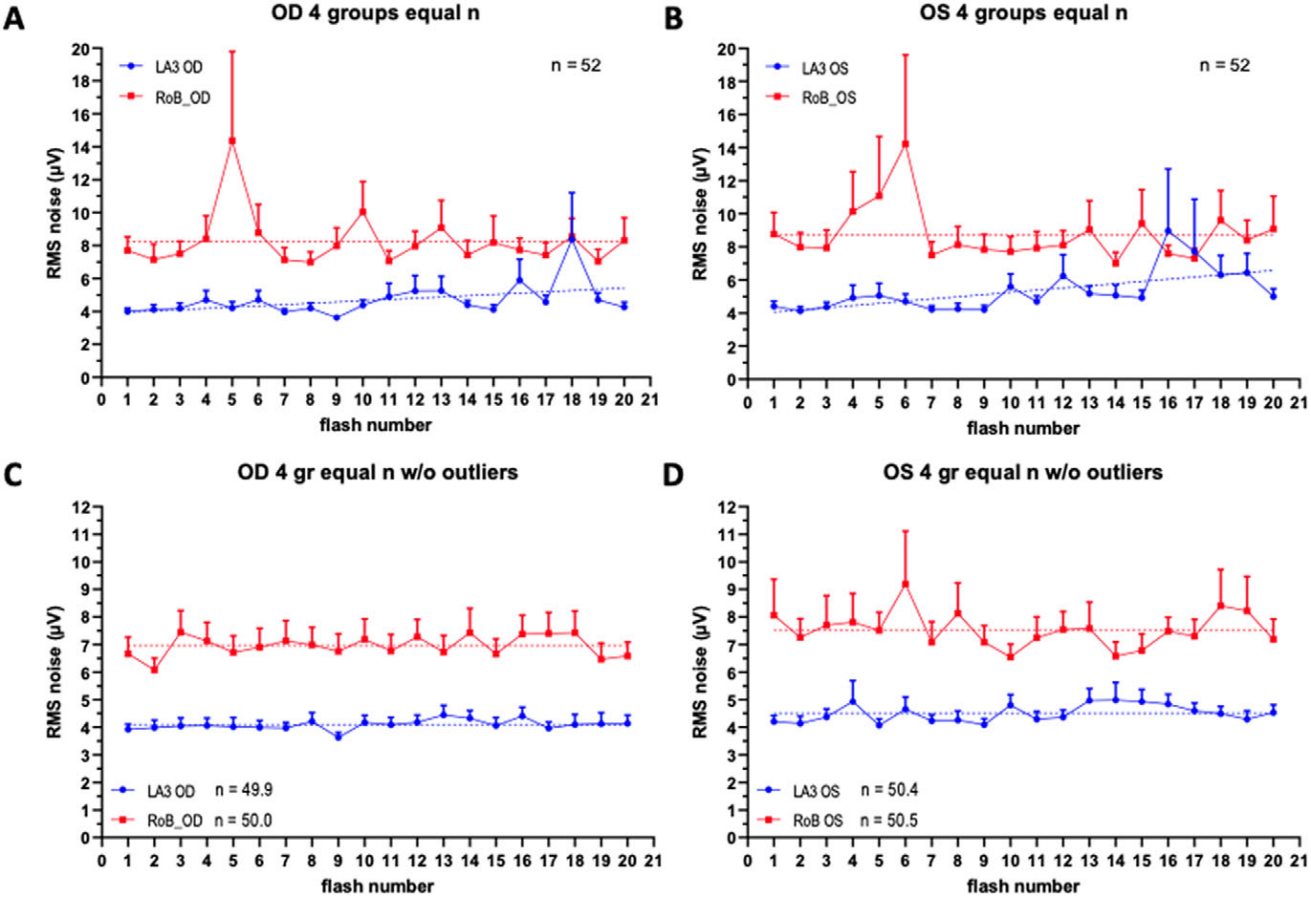
Equal size (n = 13/group) group analysis of 20 individual responses. Aggregated data from all 4 groups are shown (n = 52). Top panels: average RMS values from right eyes (left panel) and left eyes (right panel); Bottom panels: average RMS values with outliers eliminated from right eyes (left panel) and left eyes (right panel). Data points represent mean values average + SEM; horizontal dotted lines indicate a linear regression model fit to the data.

To explore further whether temporal variability of the pre-stimulus noise exists during collection of individual runs within the course of an ERG recording, we plotted mean values of RMS for LA3 and RoB conditions against consecutive flash number. A graphical representation of this analysis is shown in Figure 4A–B. High temporal variability observed at the beginning of the period for RoB responses and towards the end of the period for LA3 responses was suspected to be due to the presence of artifacts. This was confirmed after application of an outlier identification analysis and graphing of the cleaned data without outliers (Figure 4C–D). Overall, outliers were detected in ~3.5% of all signals (LA3 right eyes: 4.1%, LA3 left eyes: 3.2%, RoB right eyes: 3.9%, RoB left eyes: 3.0%). Removing outliers also eliminated signs of temporal variability and resulted in linear regression fits of the individual data for both LA3 and RoB having slopes all non-significantly different from 0 (F-test, *p* > 0.05).

As a further step in the evaluation of differences between the LA3 and RoB data, a mixed-effects model (REML) analysis was conducted on the cleaned datasets. When comparing both right and left eye datasets, the column factor (indicating differences between corresponding LA3 versus RoB records) was highly significant (*p* < 0.0001) and the average difference between predicted means was 2.91 ± 0.46 μV for right eyes and 3.0 ± 0.68 μV for left eyes, while the ratio between the predicted means was 1.71 for right eyes and 1.67 for left eyes.

These results indicate that RoB stimulation results in significantly increased baseline bioelectrical activity prior to not only the initial flash, but also the subsequent flashes throughout the ERG test.

### Effect on the rate of unambiguous PhNR component identification

**Figure 5.**
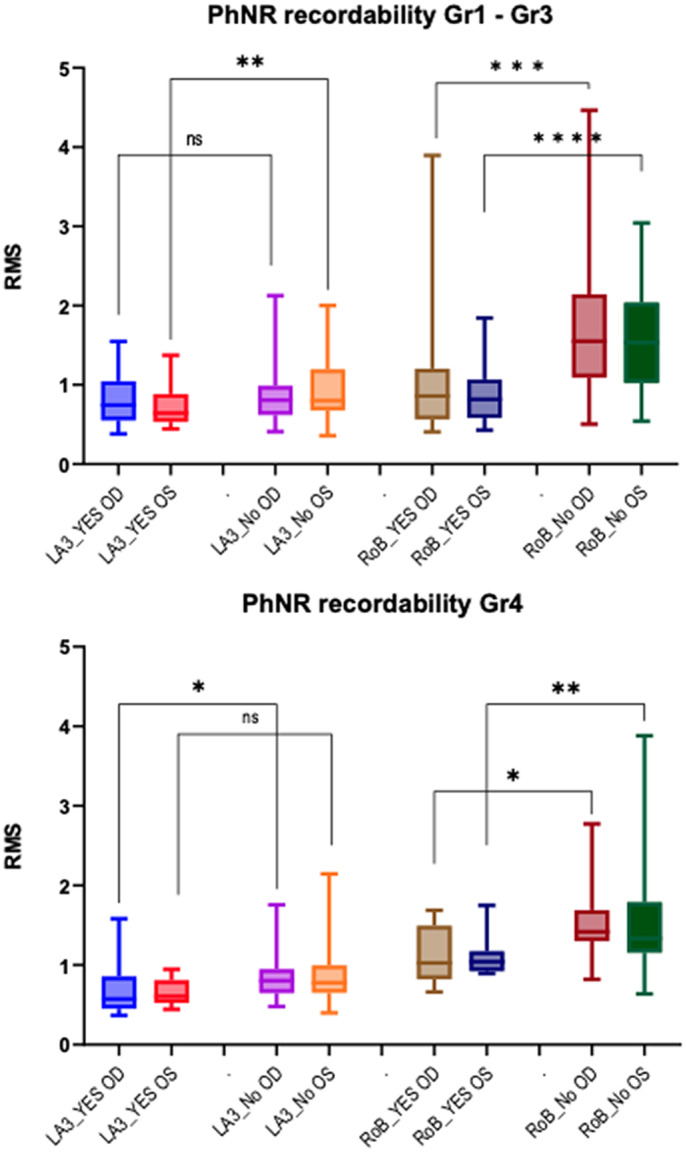
Pairwise comparisons of RMS values based on PhNR recordability. For Gr1–3 (top panel) and Gr4 (bottom panel). YES OD – PhNR identifiable in right eye; YES OS – PhNR identifiable in left eye; NO OD – PhNR not identifiable in right eye; NO OS – PhNR not identifiable in right eye. Statistical significance (Wilcoxon matched-pairs signed rank test): ns – not significant; * - *p* < 0.05; ** - *p* < 0.01; *** - *p* < 0.001; **** - *p* < 0.0001.

A summary of the results from the analysis of PhNR recordability in this dataset is presented in Figure 5 and Supplementary Table 1. Overall, the rate of presence of signals with clearly (unambiguously) identifiable PhNR components in this dataset was below 50% (33–41% for LA3 and 38–47% for RoB). For ERG signals recorded under the LA3 condition, there was not much difference between the median RMS values of the pre-stimulus noise whether the PhNR was clearly identifiable or not (range 0.6–0.8). In contrast, the median RMS values of pre-stimulus noise where PhNR was identifiable under RoB conditions were 1.3–1.9 times smaller compared to signals where PhNR was not identifiable; specifically, for the aggregated data Gr1–Gr3, the difference was highly significant (*p* < 0.001), while for Gr4, the difference was also significant but less pronounced (*p* < 0.05).

### Frequency spectrum of the signal

The frequency spectrum of the signal was evaluated for all records in Gr1–3 and the corresponding mean frequency spectra in the region 0 to 625 Hz (first 1/3 of the spectrum, containing ~2/3 of the energy) recorded under LA3 and RoB are shown in Supplementary Figure 2. It is clear from the data presented that, although the shape of the spectra recorded under the two conditions was similar, the area under the curve of the FFT magnitude of the signal recorded under RoB was ~1.5–1.7 times larger compared to the one recorded under LA3 (Supplementary Table 2).

As the data for Gr4 were obtained with different equipment and sampled with a different sampling rate (2000 Hz versus 3750 Hz for Gr1–3), they were analyzed separately but with a similar approach as with the other data. For comparison with Gr1–3, the mean frequency spectra for Gr4 in the same region (0 to 625 Hz) are shown in Supplementary Figure 3. As with Gr1–3, the RoB area under the curve in the part of the spectrum shown was ~1.7 times larger compared to LA3 and the ratio RoB/LA3 remained very close to the mean ratios obtained from the analysis of RMS (Table 2).

During the process of this analysis, it was noted that some of the FFT magnitude values were much higher than others which prompted an additional analysis for identifying outliers. Although the elimination of outliers changed the shape of the frequency spectrum (Supplementary Figures 4 and 5), the ratio RoB/LA3 of area under the curve underwent little change (Supplementary Table 2).

The different shapes of the frequency domain spectra prompted a look at the peak of the frequency spectrum in each group. This was determined as part of the area under the curve analysis in Prism and the results are summarized in Supplementary Table 3. As seen from the data presented in the table, in the majority of the groups, the peak occurred at frequencies below 60 Hz; only in Gr3 under the LA3 condition did the peaks occur at frequencies above 60 Hz (which became more pronounced after removing outliers). In none of the groups under RoB stimulation did the peaks occur above 60 Hz. The reason for this difference in peak occurrence is unknown and deserves further investigation. It is worth also noting that the average peak values of Gr1–Gr3 were similar to the peak values in Gr4, indicating that overall, the shape of the frequency spectrum of the pre-stimulus bioelectrical signal was similar despite the difference in equipment and stimulus parameters used (Table 3).

**Table 3.** Pairwise comparisons of median RMS values based on PhNR recordability

	Gr1–Gr3			Gr4		
	LA3	RoB	*p*-value^a^	LA3	RoB	*p*-value^a^
Yes OD	0.721	0.875	*	0.578	0.889	*
Yes OS	0.557	0.819	*	0.605	1.021	**
No OD	0.806	1.835	****	0.809	1.432	****
No OS	0.839	1.608	****	0.808	1.397	***

Abbreviations: LA3, light-adapted 3 (white flash on white background); RMS, root mean square; RoB, red flash on blue background; Yes OD, PhNR identifiable in right eyes; Yes OS, PhNR identifiable in left eyes; No OD, PhNR not identifiable in right eyes; No OS, PhNR not identifiable in left eyes.

a Wilcoxon matched-pairs signed rank test.

## Discussion

In the diverse patient population studied in this work, the baseline bioelectrical activity was significantly higher under the stimulation condition of a red flash on a blue background as compared to the LA3 condition (white flash on a white background) in all comparisons, indicating higher level of background facial muscle activity likely caused by more visual discomfort due to the blue background. These findings are consistent with anecdotal patient reports and with a recent systematic experiment on visual discomfort, which found that full-field blue light stimulation induces more visual discomfort compared to full-field red light stimulation, under both binocular and monocular viewing conditions in visually healthy participants with pharmacologically dilated pupils (Zivcevska et al., 2018). This could explain some of the differences in the quality of the signal and recordability of the ERG components, as noted in our previous observations (Tzekov et al., 2017, 2018; Abu-Samra et al., 2019). Our analysis of temporal variability of pre-stimulus bioelectrical activity clearly demonstrated that RoB mean RMS levels remain higher compared to LA3 throughout 20 individual responses for both right and left eyes, without any trend, showing no significant temporal variability. Furthermore, outlier analysis showed many more outliers removed under RoB, indicating that this recording condition leads to higher level of RMS signal, most likely due to gaze instability and/or increased facial muscle activity. This supports the results from the main analysis indicating higher baseline activity under RoB.

The differences in visual discomfort are likely caused from a difference in the perceived brightness of the white and blue background. Background is quantified photometrically by its photopic luminance and measured in photopic cd/m^2^. However, the perceived brightness may be substantially different because luminance and brightness are different entities (Lennie et al., 1993). Brightness has a stronger relative sensitivity to short wavelengths than luminance, explaining why blue backgrounds are perceived as brighter than the white backgrounds even at equal luminance. Many studies have used 10 cd/m^2^ blue background or brighter (Chen et al., 2008; Machida et al., 2008; Sustar et al., 2009; Kremers et al., 2012; Niyadurupola et al., 2013; Preiser et al., 2013; Moss et al., 2015; Kirkiewicz et al., 2016; Kundra et al., 2016); the values generated by this measurement do not correspond to the perceived brightness, which is higher for the short wavelength of the spectrum (Kokoschka & Adrian, 1985; Howett, 1986). Perceived brightness of large fields like the ones used in ERG background may even amplify the difference between luminance and brightness (Schanda et al., 2002). A possible additional role of the intrinsically photosensitive retinal ganglion cells, which are maximally sensitive to a light of about 480 nm, remains to be elucidated (Brown et al., 2012; Besenecker et al., 2016; Besenecker & Bullough, 2017). It is also important to note that the study by Rangaswamy et al. (2007), which proposed that RoB conditions were superior in elucidating PhNR, was performed on anesthetized monkeys, where visual discomfort does not play a role.

Additionally, some patients may be more sensitive to blue light that to white light. In migraine patients, full-field flashes of blue light increased more frequent throbbing and muscle tenderness and a spread of headache from its original site compared to equivalent luminance flashes of white light (Noseda et al., 2016).

Another reason for such a difference may be the different time of adaptation to the background illumination: >10 min for LA3 and > 2 min for RoB. Some studies have shown that time of adaptation plays a role in the comfort level associated with different colors and light intensities indicating that it takes several minutes for the comfort level to improve (Takahashi & Misawa, 2015; Fotios, 2017); however, it has to be kept in mind that these studies were done on healthy volunteers and using a natural pupil. To the best of our knowledge, this aspect of the problem has not been studied in a diverse patient population or pharmacologically dilated pupil. The time of adaptation to the background illumination is not specified in the ISCEV extended PhNR protocol (Frishman et al., 2018); perhaps studies should be conducted, and this issue should be addressed in future revisions of this document to mitigate any patient discomfort during ERG testing.

The difference in brightness and the effect of time of adaptation may have also been accentuated by the order of administration of the two conditions. RoB was always administered after the administration of LA3 and 30 Hz flicker conditions. To the best of our knowledge, such an order effect has not been studied and the magnitude of this effect, if present, remains unknown.

The spectral density of the pre-stimulus bioelectrical signal recorded in the current study was an interesting finding. Largely, the peak of the power density spectrum was close, but not equal, to 60 Hz, the mainframe power frequency in the facility where the study was conducted. Overall, the shape of the power spectrum recorded in this study corresponds to the power spectrum of the spontaneous surface EMG activity recorded from m. orbicularis oculi in healthy volunteers (van Boxtel, 2001). Therefore, the content of the pre-stimulus background bioelectrical activity could have a complex origin, likely incorporating mostly background eyelid muscle activity, but also mainframe power interference, intrinsic noise of the recording system, etc.

Our analysis of the current dataset shows that the effect of the level of pre-stimulus signal on the quality of the post-stimulus signal, as evaluated by the ability to identify an unambiguous PhNR response, was more pronounced for the RoB condition, as compared to the LA3 condition. This is in line with the other findings of this study, as discussed above. It has to be noted that, in general, the timing of the occurrence of the PhNR peak, typically at 60–70 ms post-flash, coincides with the latency of the photic blink reflex (Rushworth, 1962; Hackley & Johnson, 1996) and the photomyoclonic reflex (Johnson & Massof, 1982), artifacts that could interfere with the reliable identification and accurate measurement of the PhNR.

One limitation of the current study is the sex disbalance of the population, as only ~31% of the patients were male. This probably reflects the established notion that women in general are at greater risk of vision loss (Zambelli-Weiner et al., 2012) and therefore more likely to be referred for ERG testing. Although no sex differences are reported in the perception of white light (Rammsayer & Troche, 2012), it has been demonstrated that females are more sensitive to the Helmholtz-Kohlrausch effect, most pronounced in the blue end of the spectrum (Foutch & Bassi, 2020).

Another limitation of the study is the unequal number of patients in each group. Thus, Gr1 consisted of only 13 patients, less than half compared to the number of patients in the other groups. Nevertheless, the fact that the results from this group generally confirm the findings obtained in the other groups adds confidence to our conclusions.

Overall, based on results from a sizeable heterogenous patient population, this work suggests that a pre-stimulus bioelectrical activity level recorded under the typical white background used for the LA3 condition is lower compared to one recorded under a blue background (RoB condition) of 10 cd/m^2^ or higher, which likely reduces patient visual discomfort and lead to a decreased SNR in RoB conditions despite having larger amplitudes. Further work is needed to establish an RoB background luminance level that would produce a comparable perceived brightness and, correspondingly, a similar level of visual discomfort.

## Supporting information

Tsay et al. supplementary material Tsay et al. supplementary material

## Supplementary material

The supplementary material for this article can be found at http://doi.org/10.1017/S0952523823000032.
