# Pre-stimulus bioelectrical activity in lightadapted ERG under blue versus white background – CORRIGENDUM

**DOI:** 10.1017/S0952523823000044

**Published:** 2024-01-05

**Authors:** Katherine Tsay, Sara Safari, Abdullah Abou-Samra, Jan Kremers, Radouil Tzekov

The authors regret that this article was published with a misspelling in an author’s name: ‘Abdullah Abu-Samra’ should be ‘Abdullah Abou-Samra’. This has been updated in the original article.
