# Supplementary material for: Pre-stimulus bioelectrical activity in light-adapted ERG under blue versus white background
Source: Vis Neurosci. 2023 Dec 13;40:E004. doi: 10.1017/S0952523823000032 (PMC11016353; doi:10.1017/S0952523823000032)
Supplement: Tsay et al. supplementary material [file S0952523823000032sup001.docx]

**Supplementary Information**

for

**Tsay et al. “Pre-stimulus bioelectrical activity during photopic ERG under blue vs. white background”**

**Supplementary Fig. 1** Representative traces of signals from each of the four groups with PhNR clearly identifiable (left panels) and with PhNR not clearly identifiable (right panels). In each panel an LA3 response (blue trace) and a corresponding RoB response recorded from the same patient (red trace) are shown. From top to bottom: example from Group 1, Group 2, Group 3, and Group 4. Individual traces were shifted vertically for clarity.

**Supplementary Table 1.** Recordability of PhNR based on levels of pre-stimulus signal. The median values of RMS of pre-stimulus signal are shown for traces where PhNR was either clearly identifiable (PhNR Yes) or not (PhNR No).

|  | | LA3 | | | | RoB | | | |
| --- | --- | --- | --- | --- | --- | --- | --- | --- | --- |
|  |  | **Right Eyes** | | **Left Eyes** | | **Right Eyes** | | **Left Eyes** | |
|  |  | PhNR Yes | PhNr No | PhNr Yes | PhNr No | PhNr Yes | PhNr No | PhNr Yes | PhNr No |
| Gr1-3 | **Median** | 0.75 | 0.81 | 0.64 | 0.80 | 0.86 | 1.55 | 0.82 | 1.54 |
|  | **n** | 29 | 41 | 26 | 39 | 28 | 37 | 21 | 41 |
|  | **Recordability** | 41.4% | | 40.0% | | 43.1% | | 33.9% | |
| Recordability (Conditions) | | 40.7% | | | | 38.5% | | | |
| Gr4 | **Median** | 0.57 | 0.80 | 0.61 | 0.78 | 1.03 | 1.42 | 1.04 | 1.33 |
|  | **n** | 15 | 25 | 12 | 29 | 20 | 19 | 17 | 23 |
|  | **Recordability** | 37.5% | | 29.3% | | 51.3% | | 42.5% | |
| Recordability (Conditions) | | 33.4% | | | | 46.9% | | | |

**Supplementary Fig.** **2** Frequency spectrum of the signals for LA3 and RoB in Gr1 to 3 with outliers. The signal was zero padded to 512 samples to increase frequency resolution and windowed with a Hamming window to reduce transitioning artifacts. Mean values and SEM of FFT magnitudes are shown for right (black color) and left (red color) eyes separately. Area under the curve based on mean values is shown in each inset. Left panels – LA3 spectrum, right panels – RoB spectrum

**Supplementary Fig. 3** Frequency spectrum of the signals for Gr4 with outliers. Other designations same as in Supplementary Fig. 2

**Supplementary Fig. 4** Frequency spectrum of the signals for LA3 and RoB in Gr1 to 3 with outliers removed. Outliers were identified and removed for each frequency by the ROUT method (Q = 1%). Mean values and SEM of FFT magnitudes are shown for right (black color) and left (red color) eyes separately. Area under the curve based on mean values is shown in each inset. Left panels – LA3 spectrum, right panels – RoB spectrum.

**Supplementary Fig. 5** Frequency spectrum of the signals for Gr4 without outliers. Outliers were identified and removed for each frequency by the ROUT method (Q = 1%). Other designations same as in Supplementary Fig. 4

**Supplementary Table 2.** Area under the curve (AUC) of the FFT spectrum (0 – 625 Hz) based on mean values for LA3 and RoB in Gr1 to 4 with and without outliers.

|  |  | | **AUC with outliers** | | **AUC without outliers** | |
| --- | --- | --- | --- | --- | --- | --- |
|  |  | | **OD** | **OS** | **OD** | **OS** |
| **Gr1** | **LA3** | | 7586 | 7840 | 6075 | 6679 |
|  | **RoB** | | 10960 | 11465 | 10583 | 10176 |
|  | **RoB/LA3 ratio** | | 1.44 | 1.46 | 1.74 | 1.52 |
| **Gr2** | **LA3** | | 5587 | 6062 | 5500 | 5824 |
|  | **RoB** | | 13665 | 12814 | 11601 | 10800 |
|  | **RoB/LA3 ratio** | | 2.45 | 2.11 | 2.11 | 1.85 |
| **Gr3** | **LA3** | | 6355 | 8165 | 6075 | 6679 |
|  | **RoB** | | 8825 | 8668 | 7646 | 7950 |
|  | **RoB/LA3 ratio** | | 1.39 | 1.06 | 1.26 | 1.19 |
| **Mean RoB/LA3 Ratio**  **Gr1-Gr3** | | | 1.76 | 1.55 | 1.70 | 1.52 |
| **Gr4** | | **LA3** | 2812 | 2821 | 2680 | 2623 |
|  |  | **RoB** | 4805 | 4830 | 4655 | 4358 |
|  |  | **RoB/LA3 ratio** | 1.71 | 1.71 | 1.74 | 1.66 |

**Supplementary Table 3.** Summary of the peak values of the bioelectrical signal in the frequency domain. The peaks were determined by Prism as part of the area under the curve analysis. All values are shown in Hz.

|  | Light-adapted 3 ERG | | | |  | Red flash on blue background | | | |
| --- | --- | --- | --- | --- | --- | --- | --- | --- | --- |
|  | *With outliers* | | *Without outliers* | |  | *With outliers* | | *Without outliers* | |
|  | **OD** | **OS** | **OD** | **OS** |  | **OD** | **OS** | **OD** | **OS** |
| Gr1 | 14.6 | 0.0 | 51.3 | 51.3 |  | 58.6 | 44.0 | 58.6 | 44.0 |
| Gr2 | 51.3 | 44.0 | 44.0 | 44.0 |  | 44.0 | 36.6 | 36.6 | 36.6 |
| Gr3 | 73.2 | 51.3 | 80.6 | 95.2 |  | 44.0 | 58.6 | 58.6 | 58.6 |
| Average  Gr1-Gr3 | 46.4 | 31.8 | 58.6 | 63.5 |  | 48.9 | 46.4 | 51.3 | 46.4 |
| Gr4 | 46.9 | 43.0 | 46.9 | 43.0 |  | 46.9 | 39.0 | 46.9 | 43.0 |
